# Supramolecular Multiple Stimuli-Responsive Conductive Hydrogel for Flexible Sensing

**DOI:** 10.3390/gels12050392

**Published:** 2026-05-02

**Authors:** Zexing Deng, Litong Shen

**Affiliations:** 1 College of Materials Science and Engineering, Xi’an University of Science and Technology, Xi’an 710054, China; 24211225047@stu.xust.edu.cn; 2 Frontier Institute of Science and Technology, Xi’an Jiaotong University, Xi’an 710049, China

**Keywords:** conductive self-healing hydrogel, stimuli-responsive properties, supramolecular interactions, flexible sensor

## Abstract

Self-healing conductive hydrogels have attracted considerable interest in recent research due to their applications in biomedical and electronic fields. The design and preparation of a functional self-healing conductive hydrogel that features multiple stimuli-responsive properties, adhesion, and tunable mechanical characteristics for a wearable electronic sensor is highly anticipated. In this work, we proposed a hydrogel sensor through free radical polymerization by using host molecule acryloyl-β-cyclodextrin (AC-β-CD), guest molecule of acryloyl-1-adamantanamine (AC-AD), N-isopropylacrylamide (NIPAM), and conductive reduced graphene oxide/β-CD (rGO-CD). The chemical and physical structure, conductivity, de-swelling/swelling behavior, photothermal behavior, mechanical performance, adhesive performance, injectable performance, and self-healing performance of the resultant hydrogels were comprehensively investigated. Human motion detection and cytocompatibility test of hydrogel further demonstrated its potential for wearable electronics applications. Overall, this supramolecular conductive hydrogel might open a new sight to develop a multifunctional flexible sensor.

## 1. Introduction

Flexible sensors based on hydrogel are capable of transferring stimuli including heat, pressure, light, and voice into recordable electrical outputs to reflect the status of environments [1,2,3]. They have attracted great interest in scientific research for their possible application in electronic skin [4,5,6], human–machine mutual interactions [7,8,9], and healthcare diagnosis [10,11,12]. Therefore, developing suitable hydrogels with electrical conductivity, multiple stimuli-responsive properties, self-healing performance, adhesive ability, injectable performance, and biocompatibility for flexible sensor applications is a hot research spot in recent years [13,14,15].

Among these properties of hydrogel, the conductivity is essential for flexible sensor applications [16,17,18]. Conductive hydrogels have been developed due to their combination of metal-like electrical conductivity and the flexible mechanical properties of hydrogels, integrating both electrical and mechanical advantages. Furthermore, the incorporation of conductivity might endow hydrogels with multiple stimuli-responsivity (near-infrared light and strain/stress stimuli-responsivity), thereby broadening their sensing applications [19,20].

Nevertheless, traditional conductive hydrogels often lack self-healing performance, thereby limiting their application in dynamic sensing environments, such as human motion detection. The concept of self-healing was inspired by the healing process of natural organisms, allowing a material to recover its functionality after damage [21,22]. Self-healing performance of hydrogel means that the hydrogel can repair its structure and function to its original state through external stimulus or its own intermolecular interaction after being damaged, thereby extending the lifespan of the hydrogel and improving its reliability. To design autonomous self-healing hydrogels, a common approach involves the use of intermolecular interaction-based bonding systems, wherein crosslinking is achieved through dynamic chemical bonds (e.g., Schiff-base bond [23], boric acid ester bond [24], disulfide bond [25]) and/or dynamic physical interactions, such as hydrogen bond [26], metal–ligand coordination interactions [27], and host–guest interactions [28]). Among them, host–guest interactions are formed between two molecules, one of which is the host molecule (β-cyclodextrin, cucurbituril, etc.) and the other is the guest molecule (1-adamantanamine, aromatic molecules, etc.). The guest molecule can enter the hydrophobic cavity of the host molecule to generate the host–guest interaction. By way of illustration, Scherman and co-workers developed a self-healing conductive hydrogel employing host molecule cucurbituril, monomer acrylamide and the guest molecule 1-benzyl-3-vinyl imidazole [29]. The self-healing hydrogel constructed via host–guest interactions exhibits super stretchability, stretching to 100 times its original length, and super toughness, withstanding up to 2000 times its own weight. Recently, β-cyclodextrin (β-CD) as a host molecule and 1-adamantanamine (AD) as a guest molecule have been reported to establish a supramolecular hydrogel system, because β-CD and AD can be facilely chemically designed and functionalized [30,31,32].

Poly(N-isopropylacrylamide) (PNIPAM) is characterized by the presence of amide groups (–C=O–NH–) and pendant isopropyl units (–CH(CH_3_)_2_) along its backbone, with the former contributing hydrophilic character and the latter providing hydrophobic domains. Hydrogels constructed from PNIPAM undergo a pronounced volume phase transition at approximately 32 °C, and have been reported for sensing applications, such as on–off switches, owing to their pronounced thermal responsiveness [33,34]. However, traditional PNIPAM hydrogels often lack electrical conductivity, self-healing properties and adhesive properties; these drawbacks would hinder their sensor application [35]. Although some researchers have simply explored the PNIPAM-based hydrogels in human motion monitoring, the research on PNIPAM-based hydrogels in this field is not yet sufficient, and further studies are still anticipated.

In this manuscript, we demonstrate a conductive hydrogel with multiple stimuli-responsivity, self-healing ability, stretchability, adhesive performance, and cytocompatibility for flexible sensor application. Acryloyl-β-cyclodextrin (AC-β-CD), acryloyl-1-adamantanamine(AC-AD), and N-isopropylacrylamide (NIPAM) were copolymerized to synthesize the self-healing adhesive hydrogel. Conductive component rGO-CD was introduced into the hydrogel network to endow it with electrical conductivity. The chemical and physical structure, properties such as stimuli-responsivity of temperature and NIR light, mechanical property, adhesive property, and self-healing performance were tested and discussed. Moreover, the hydrogel applied in remote controllable valve and human motion detection was also developed. In summary, this hydrogel has great potential as an adhesive, self-healing, flexible sensor.

## 2. Results and Discussion

### 2.1. Preparation of the Supramolecular Conductive Hydrogels

**Figure 1:**
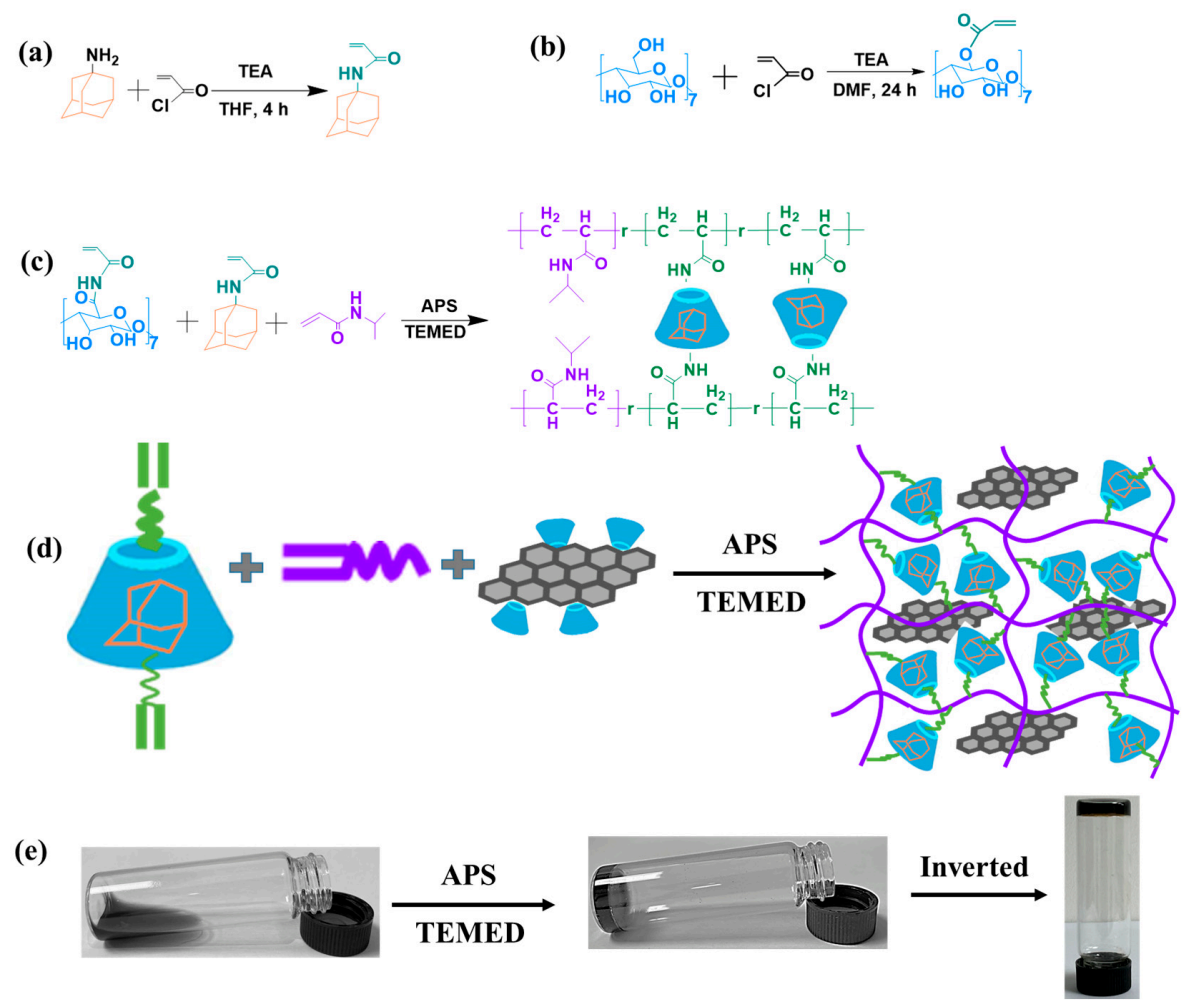
Scheme of preparation of AD-CD-PNIPAM and AD-CD-PNIPAM/rGO hydrogels: (**a**) Synthesis of AC-AD. (**b**) Synthesis of AC-CD. (**c**) Synthesis of AD-CD-PNIPAM hydrogel. (**d**) Synthesis of AD-CD-PNIPAM/rGO hydrogel. (**e**) Pictures of synthesis AD-CD-PNIPAM/rGO hydrogel.

Conductive hydrogels based on supramolecular interactions brought great interest to scientific researchers, because they could have self-healing and multiple stimuli-responsive properties, greatly expanding the application range of hydrogels [36,37,38,39]. The preparation scheme of our conductive supramolecular hydrogel is displayed in Figure 1. First, the AD and β-CD were respectively reacted with AC to obtain double carbon bond modified AC-AD and AC-β-CD (Figure 1a,b). Then, as demonstrated in Figure 1c, AC-AD and AC-β-CD were first formed into a complex of AC-AD/AC-β-CD and copolymerized with NIPAM to form the supramolecular self-healing hydrogel AD-CD-PNIPAM. The conductive component rGO-CD was formed through the self-assembly of GO and CD. Finally, the rGO-CD dispersion was introduced into the AD-CD-PNIPAM hydrogel network to obtain the conductive supramolecular hydrogel AD-CD-PNIPAM/rGO displayed in Figure 1d. When the grafting ratio of double carbon bond was close to 0.5, 1.0 and 1.5, the hydrogel was entitled as AD-0.5CD-PNIPAM/rGO, AD-CD-PNIPAM/rGO, and AD-1.5CD-PNIPAM/rGO, respectively. Figure 1e exhibits the pictures of the preparation process of the hydrogel. The monomer precursor solution could form the hydrogel after adding the ammonium persulfate (APS) and *N*,*N*,*N*′,*N*′-tetramethylethylenediamine (TEMED) initiator, indicating the successful preparation of the hydrogel.

**Figure 2:**
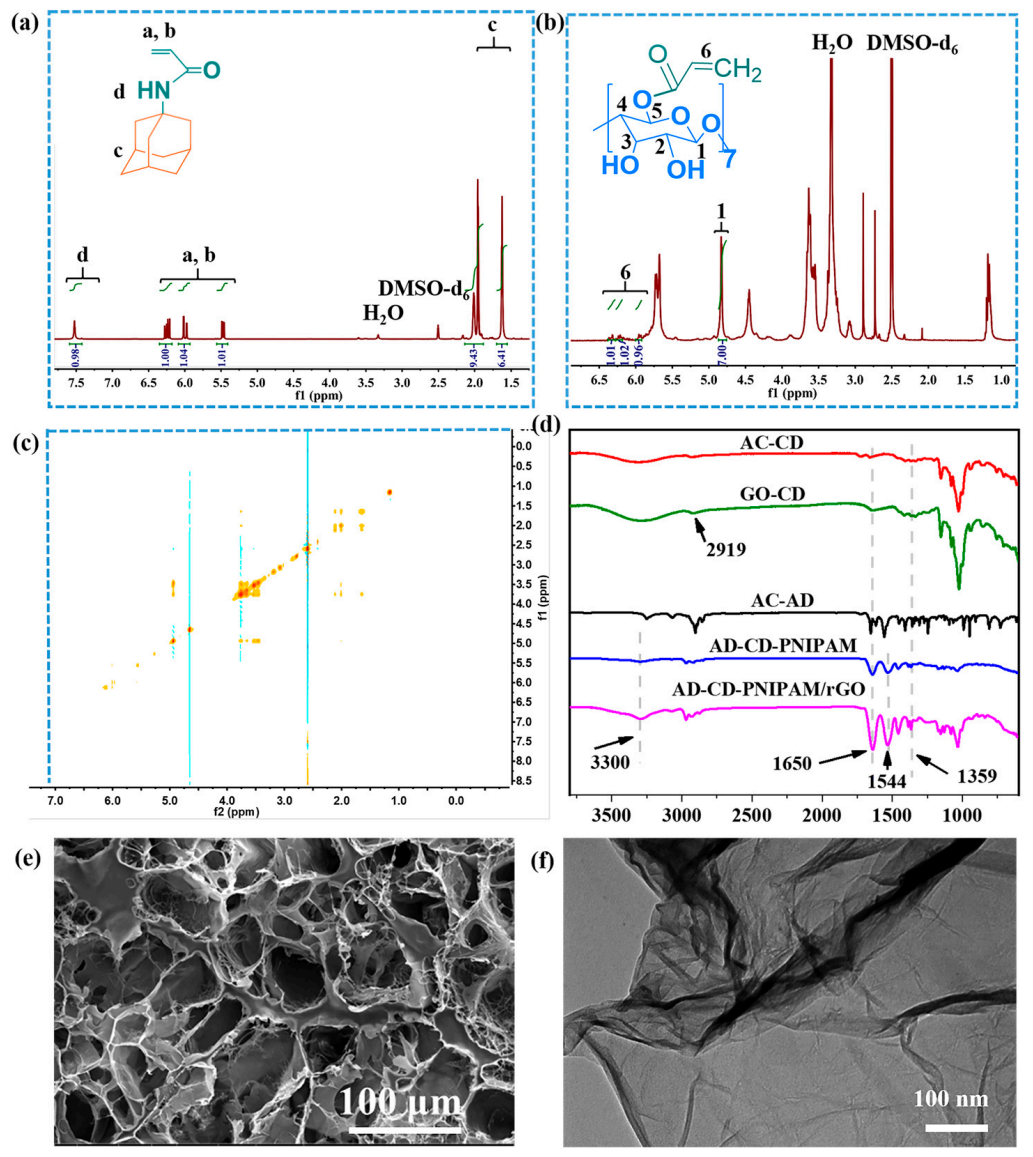
Chemical and physical structure of AC-CD, AC-AD, GO-CD, AD-CD-PNIPAM and AD-CD-PNIPAM/rGO: (**a**) ^1^H NMR spectrum of AC-AD. (**b**) ^1^H NMR spectrum of AC-CD. (**c**) 2D ^1^H NMR spectrum of AD/β-CD. (**d**) FT-IR spectra of AC-CD, AC-AD, GO-CD, AD-CD-PNIPAM and AD-CD-PNIPAM/rGO. (**e**) SEM image of AD-CD-PNIPAM/rGO hydrogel. (**f**) TEM image of rGO.

The chemical and physical structure of the supramolecular hydrogel was first investigated through FT-IR, ^1^H NMR and SEM. The ^1^H NMR spectra of AC-AD and AC-β-CD are depicted in Figure 2a,b and Figure S1. The chemical shift in the double carbon bond was shown at ~5.5–6.5 ppm (AC-AD) and ~5.8–6.5 ppm (AC-CD), respectively, indicating that the double carbon bond was grafted onto the AD and β-CD successfully. The 2D ^1^H NMR spectrum for the AD/CD mixture, provided in Figure 2c, confirms the occurrence of supramolecular association involving CD and AD. The FT-IR results of sample AC-CD, GO-CD, AC-AD, AD-CD-PNIPAM, and AD-CD-PNIPAM/rGO are displayed in Figure 2d. In the spectrum of AC-CD-PNIPAM, the bands appearing at 3421 cm^−1^ and 1632 cm^−1^ are characteristic of N–H stretching and the amide carbonyl moiety of NIPAM, while the signal centered at 1050 cm^−1^ originates from C–O–C vibrational modes [40]. Furthermore, the peak at 808 cm^−1^ of the AC-CD curve assigned to a double carbon bond disappeared in the curve of AC-CD-PNIPAM, which indicated that AC-CD was copolymerized with NIPAM. A peak observed at 2919 cm^−1^ of the GO-CD curve corresponded to the stretching vibration of C-H, while the typical peak of C=O of the GO-CD curve at 1710 cm^−1^ disappeared, indicating the GO was reduced to rGO.

The physical structure of the hydrogel was confirmed by the SEM images displayed in Figure 2e. The AD-CD-PNIPAM/rGO hydrogel exhibited a macro-porous structure with an average pore size of around 33.4 µm, characterized by the ImageJ software (version of 1.54) (Figure S2), which could allow large deformation of the hydrogel. As illustrated in Figure 2f, the rGO exhibited a typical smooth and sliced structure [41] observed by TEM, which could provide electrical conductivity for the hydrogel.

### 2.2. Thermal-Responsive Behavior of Hydrogels

**Figure 3:**
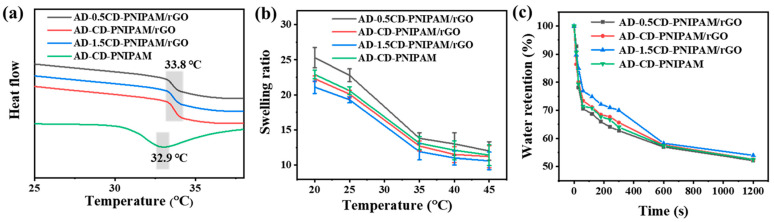
Thermal-responsive behavior of PNIPAM-based supramolecular hydrogels: (**a**) DSC curve of hydrogels. (**b**) Swelling ratio of hydrogels at different temperatures. (**c**) Deswelling kinetics of hydrogels at 50 °C.

PNIPAM-based hydrogels exhibit thermal-responsive behavior, swelling when the temperature is below their volume phase transition temperature (VPTT) and shrinking when the temperature exceeds this threshold [42,43]. Such thermal-responsive characteristics, including VPTT, temperature-dependent swelling ratio, and deswelling kinetics, are shown in Figure 3. It was obvious that all the hydrogels containing rGO possessed the same VPTT around 33.8 °C, while the AD-CD-PNIPAM hydrogel disclosed a VPTT of 32.9 °C as displayed in Figure 3a, and the VPTT was close to the lower critical solution temperature (LCST) of pure PNIPAM, indicating negligible influence on the VPTT of the hydrogel. This is because most of the hydrogel consisted of PNIPAM. Figure 3b demonstrates the temperature-dependent swelling ratio (from 21.1 to 10.6 for AD-0.5CD-PNIPAM/rGO, 22.3 to 11.2 for AD-CD-PNIPAM/rGO, 25.3 to 12.0 for AD-1.5CD-PNIPAM/rGO, and 22.9 to 11.4 for AD-CD-PNIPAM, respectively) of the hydrogel from 20 °C to 45 °C. There was a sharp decrease in swelling ratio after the temperature increased from 25 °C to 35 °C because of the phase transition behavior of PNIPAM-based hydrogel. With the increase in carbon–carbon double bond grafting ratio, there was a decrease in swelling ratio due to the increased crosslinking intensity of the hydrogel. The shrinking kinetics at 50 °C of the hydrogel is shown in Figure 3c. All the hydrogels demonstrated similar deswelling behavior, which could lose ~42–43% of water in just 600 s. After deswelling for 1200 s, hydrogels could lose ~46–48% of water, demonstrating a suitable deswelling rate. These results indicated that our PNIPAM-based hydrogels exhibited remarkable thermal-responsive properties, which are beneficial for their thermal-responsive sensor utilizations.

### 2.3. Adhesive and Photothermal Properties of Hydrogels

**Figure 4:**
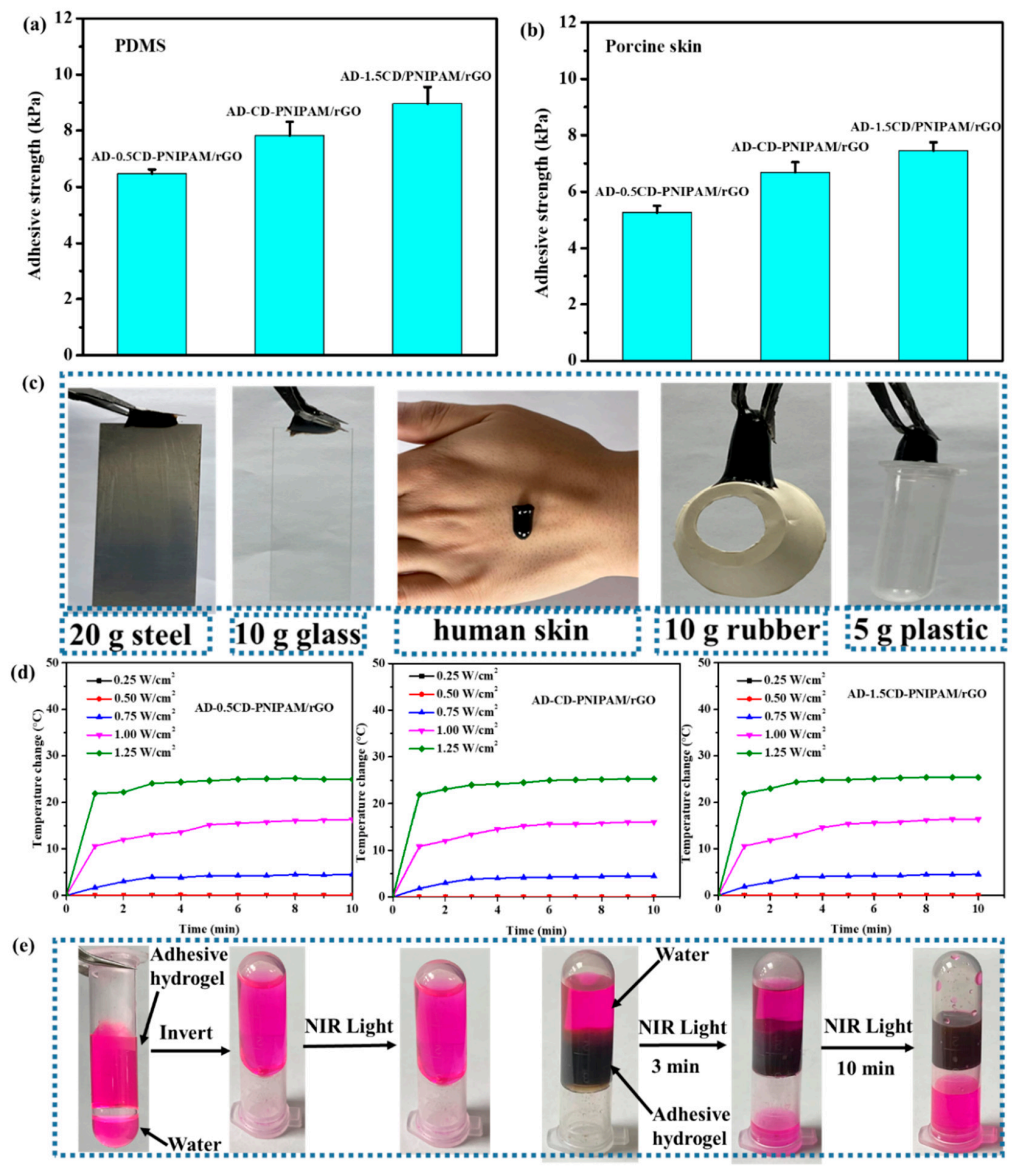
Photothermal and adhesive properties of AD-CD-PNIPAM/rGO hydrogels. Adhesive strength of AD-CD-PNIPAM/rGO hydrogels using (**a**) PDMS and (**b**) porcine skin as substrates. (**c**) Photographs of AD-CD-PNIPAM/rGO hydrogels adhere to different substrates. (**d**) Temperature change in AD-CD-PNIPAM/rGO hydrogels under different NIR light power intensity. (**e**) Auto-valve application of hydrogels under NIR light exposure.

The adhesive ability of hydrogel is beneficial for the wearable sensor application [44,45]. For hydrogels without adhesive performance, they require additional fixing devices, which makes the application process complicated. In addition, it is inevitable that there would be friction and interfacial delamination between the fixing device and hydrogel during extended operation, leading to a reduction in the detection sensitivity of the hydrogel. We further investigated the adhesive performance of the AD-CD-PNIPAM/rGO hydrogels. As demonstrated in Figure 4a,b, the AD-CD-PNIPAM/rGO hydrogels demonstrated decent adhesive strength from 6.5 kPa to 9.0 kPa for polydimethylsiloxane (PDMS) substrate and 5.2 kPa to 7.5 kPa for porcine skin substrate, which are comparable to our previously published work [46]. Good adhesive performance of hydrogels was attributed to hydrogen bonding and intermolecular interactions of PNIPAM. The adhesive strength was increased with the increase in the double carbon bond grafting ratio because it would increase the mechanical strength of the hydrogel. Figure 4c displays that the hydrogel could adhere to different organic and inorganic substrates such as steel, glass, human skin, rubber and plastic, indicating good adhesive ability of the hydrogel. The good adhesive performance was beneficial for its sensor application.

Usually, rGO exhibits photothermal properties by efficiently absorbing near-infrared (NIR) light and converting it into heat. It is reasonable to investigate the photothermal behavior of hydrogels after the incorporation of rGO into the hydrogel network. The photothermal effect mechanism of rGO is described as follows: rGO can generate localized surface plasmon resonance after absorbing NIR light. And energy generated by the resonance can be converted into heat energy, resulting in the photothermal effect. The temperature change in the AD-CD-PNIPAM hydrogels after exposure to NIR light was demonstrated in Figure 4d. The hydrogels did not show a photothermal effect when the NIR light power was below 0.5 W/cm^2^. All the hydrogels demonstrated similar temperature change curves after absorbing NIR light with a power of 1.25 W/cm^2^. The temperature change could reach 25.0 °C (AD-0.5CD-PNIPAM/rGO)-25.4 °C (AD-1.5CD-PNIPAM/rGO) in just 10 min, which indicated efficient photothermal conversion efficiency. All the hydrogels displayed similar temperature change because the photothermal property was endowed by rGO. As the NIR light power increased from 0.75 W/cm^2^ to 1.25 W/cm^2^, there was an increase in temperature change from 4.6 °C to 25.0 °C (AD-0.5CD-PNIPAM/rGO). The final temperature was significantly higher than the VPTT of the hydrogel, and hydrogels can demonstrate deswelling behavior after NIR light exposure. Inspired by this nature, we designed an auto-valve shown in Figure 4e. As a control, the white AD-CD-PNIPAM hydrogel could not show photothermal behavior, and water remained in the EP tube after NIR light exposure. On the contrary, the black AD-CD-PNIPAM/rGO hydrogel could exhibit shrinking behavior under NIR light exposure, resulting in the release of water. With the increase in exposure time, all of the water could be released from top to bottom gradually. The position of the AD-CD-PNIPAM/rGO hydrogel was not changed after NIR light exposure because the hydrogel could adhere to the EP tube. This result indicated that the AD-CD-PNIPAM/rGO hydrogel could be utilized as NIR light-controllable valves.

### 2.4. Mechanical Properties of Hydrogels

**Figure 5:**
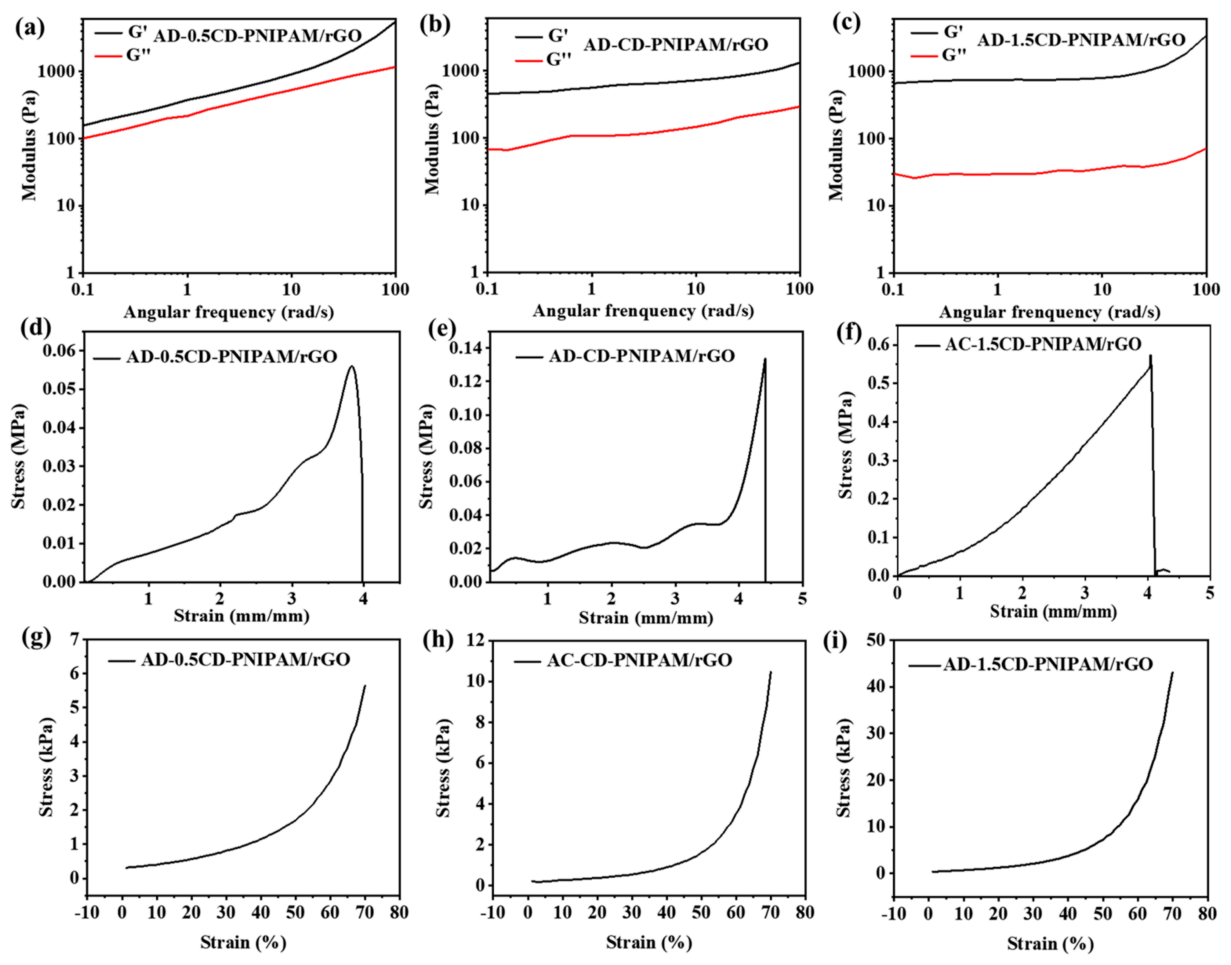
Mechanical properties of AD-CD-PNIPAM/rGO hydrogels: (**a**–**c**) Rheology curves of hydrogels. (**d**–**f**) Tensile stress–strain curves of hydrogels. (**g**–**i**) Compressive curves of hydrogels.

The hydrogels require suitable mechanical properties such as flexible, stretchable and compressible performance for dynamic sensing applications. The rheology, stretchable, and compressible behavior of the AD-CD-PNIPAM/rGO hydrogels are displayed in Figure 5. The rheology curves reflected the viscoelasticity of the AD-CD-PNIPAM/rGO hydrogel. As revealed by the rheological data, the storage modulus (G′) dominated over the loss modulus (G″) throughout the tested angular frequency window of 0.1 to 100 rad/s, demonstrating that the samples can maintain a gel state in a suitable angular frequency (Figure 5a–c). With the increase in the double carbon bond grafting ratio on CD, there was an increase in the initial storage modulus. The AD-CD-PNIPAM/rGO hydrogels could still maintain desirable flexible performance with the increase in the double carbon bond grafting ratio. This was because most parts of AD-CD-PNIPAM/rGO were made of a flexible PNIPAM chain.

Figure 5d–f demonstrate the mechanical tensile stress–strain behavior of the AD-CD-PNIPAM/rGO hydrogels. The strain at break of all the AD-CD-PNIPAM/rGO hydrogels was more than 400%, which was quite higher than traditional flexible electronics. The desirable strain value was beneficial for their application in a large deformation sensing situation, overcoming the defects of conventional flexible electronics. The double carbon bond grafting ratio on CD had a minor influence on strain at break. There was an increase in tensile strength with the increase in the double carbon bond grafting ratio on CD. The tensile strength of hydrogels was increased from ~0.06 MPa (AD-0.5CD-PNIPAM/rGO) to ~0.57 MPa (AD-1.5CD-PNIPAM/rGO) when the double carbon bond grafting ratio on CD increased from 0.5 to 1.5. The modulus was close to the soft tissues of humans, such as skin, which enhanced the compatibility of the hydrogel when it was used as a wearable sensor. The compressive curves of the AD-CD-PNIPAM/rGO hydrogels are shown in Figure 5g–i. It was obvious that all the hydrogels displayed excellent compressible mechanical properties without mechanical failure, because the axial force did not exhibit an obvious plunge during the compression process. Similarly, the compression stress at 70% strain would be improved from ~5.48 kPa (AD-0.5CD-PNIPAM/rGO hydrogel) to ~10.46 kPa (AD-CD-PNIPAM/rGO hydrogel) and ~42.83 kPa (AD-1.5CD-PNIPAM/rGO hydrogel) due to the double carbon bond grafting ratio increase. In all, these hydrogels have appropriate rheological, stretchable, and compressible properties, which facilitate their utilization as flexible sensors.

### 2.5. Self-Healing and Injectable Properties of Hydrogels

**Figure 6:**
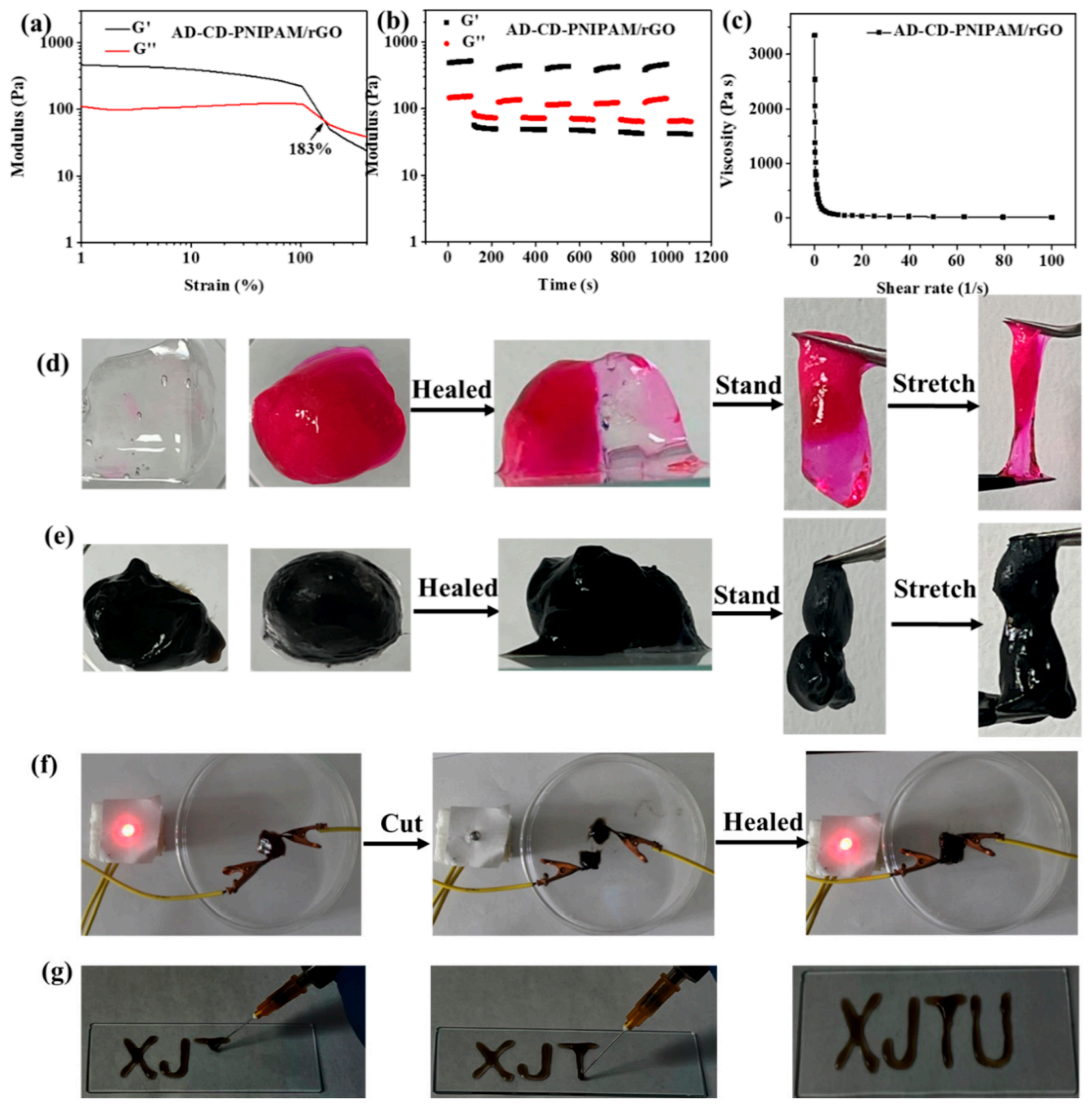
Self-healing and injectable properties of AD-CD-PNIPAM/rGO hydrogel: (**a**) Storage modulus and loss modulus of AD-CD-PNIPAM/rGO hydrogel curves versus strain. (**b**) Storage modulus and loss modulus of AD-CD-PNIPAM/rGO hydrogel at low strain (1%) and high strain (400%) mode. (**c**) Shear-thinning behavior of AD-CD-PNIPAM/rGO hydrogel. Self-healing photographs (**d**) AD-CD-PNIPAM and (**e**) AD-CD-PNIPAM/rGO hydrogels. (**f**) AD-CD-PNIPAM/rGO hydrogel served as a self-healing electronic circuit. (**g**) Injectable photographs of AD-CD-PNIPAM/rGO hydrogel.

Self-healing properties can extend the lifespan of conductive hydrogel and reduce its unreliability [47,48]. Human motion sensing and pressure sensing are dynamic processes. The structure of conductive hydrogel would be damaged under this dynamic environment and the performance of hydrogels could be weakened, which may limit their further application. Therefore, the researchers prepared a conductive hydrogel with self-healing properties for sensing applications. The self-healing property of the hydrogel is demonstrated in Figure 6. First, we investigated the strain-dependent crossover point of storage modulus and loss modulus for the AD-CD-PNIPAM/rGO sample as the strain increased from 1% to 400%, indicating the sol–gel transition state of the sample. As demonstrated in Figure 6a, the loss modulus exceeded the storage modulus when the strain was more than 183%, and the sample would undergo gel state to sol state resulted in the break of the network of hydrogel. Then, we examined the storage modulus and loss modulus under alternating high strain (400%) and low strain (1%) for five cycles as shown in Figure 6b. The sample could maintain a gel state when it was at low strain. On the contrary, the sample would turn into a sol state when it was at high strain. This result expressed that our hydrogel exhibited superior self-healing properties. The macroscopic self-healing behavior of the hydrogel is shown in Figure 6d–f: two pieces of the AD-CD-PNIPAM and AD-CD-PNIPAM/rGO hydrogels could self-heal and turn into a regular piece, and the self-healed hydrogel could withstand external force without breaking. Also, the self-healed hydrogel could illuminate LED light, indicating the self-healed electrical conductivity of the AD-CD-PNIPAM/rGO hydrogel. The macroscopic self-healing behavior further demonstrated the excellent self-healing capability of the AD-CD-PNIPAM/rGO hydrogel. This capability arises from the presence of hydrogen bonding (PNIPAM) and host–guest supramolecular interactions within the hydrogel matrix. In addition, the resultant hydrogel demonstrated a quick self-healing rate. It took approximately 10 s to self-heal due to the rich host–guest interactions and hydrogen bonding. The quick self-healing rate of the hydrogel was also verified by an alternate step-strain rheological test. The storage modulus could immediately recover to its original level when the large applied strain returned to the original small strain, indicating the quick recovery of the hydrogel network. The self-healing efficiency was assessed by the ratio of recovered storage modulus to original storage modulus. The calculated self-healing efficiency was 84.0%, further indicating decent self-healing ability.

The injectable performance of the AD-CD-PNIPAM/rGO hydrogel is also demonstrated in Figure 6c,g. As shown in Figure 6c, the hydrogel exhibited shear-thinning behavior with viscosity decreasing markedly upon increasing shear rate, indicating its injectability. Then, the hydrogel was injected into different characters shown in Figure 6g, further demonstrating the hydrogel’s excellent injectable performance. The self-healing and injectable performance were attributed to the supramolecular interactions in the hydrogel system, which were under dynamic equilibrium during the self-healing and injectable process.

### 2.6. Human Motion Sensing of Hydrogels

**Figure 7:**
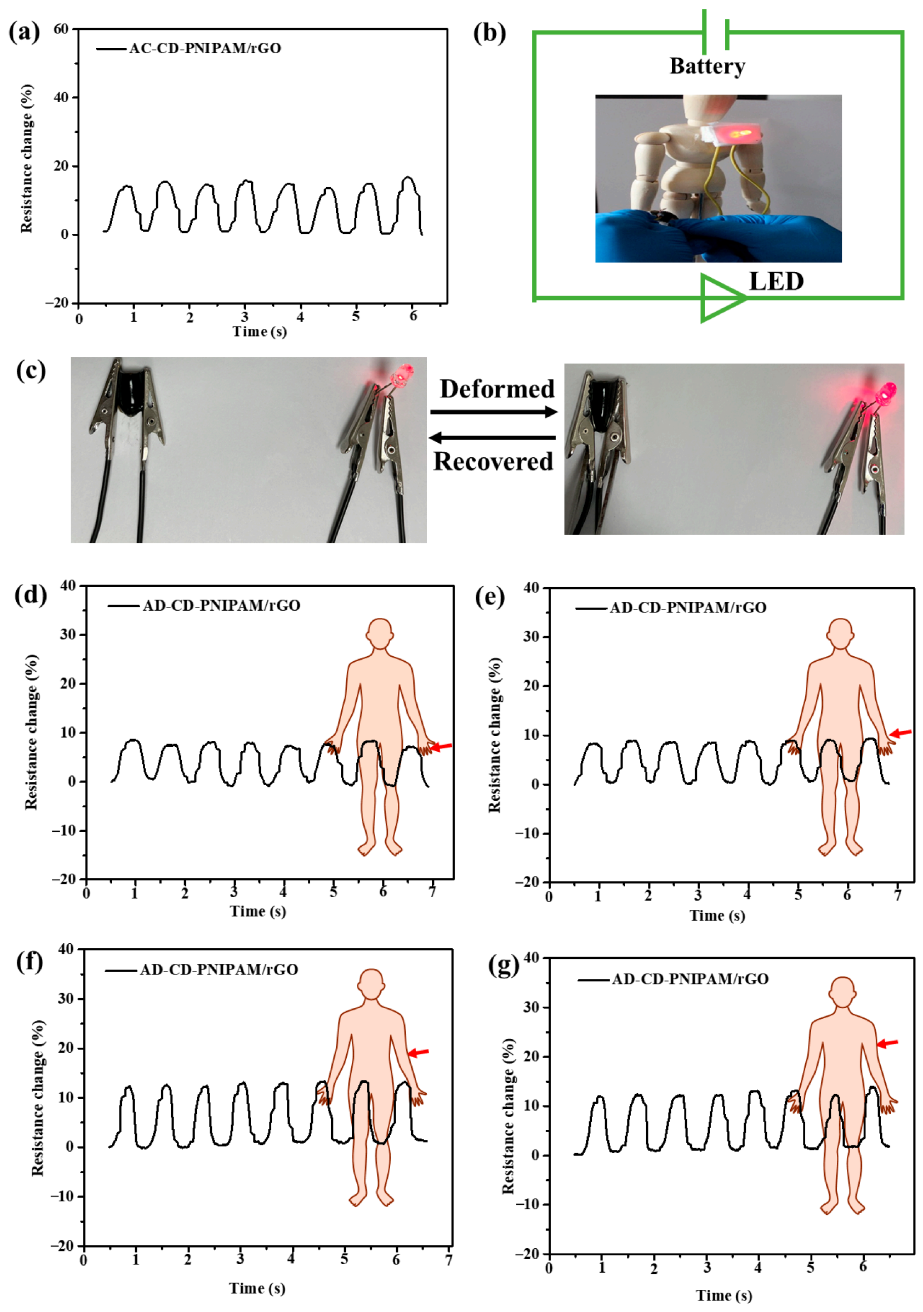
Mechanical deformation and human motion sensing application of AD-CD-PNIPAM/rGO hydrogel: (**a**) Mechanical deformation sensing curves of AD-CD-PNIPAM/rGO hydrogel. (**b**) Illustration of AD-CD-PNIPAM/rGO hydrogel for mechanical deformation sensing. (**c**) Photographs of AD-CD-PNIPAM/rGO hydrogel for mechanical deformation-sensing electronic circuit. (**d**–**g**) AD-CD-PNIPAM/rGO hydrogel for human motion detection.

Because self-healing conductive hydrogels have great potential for application in human motion detection, physiological signal tracking, artificial electronic/ionic skins, and human–machine interfacing, the design of such self-repairing materials applied to the sensing field has important research significance [49,50,51]. Self-healing conductive hydrogels served as wearable sensors for strain–stress-induced human motion detection have become a research hotspot in recent years. In view of the good electrical conductivity and flexible mechanical performance of the AD-CD-PNIPAM/rGO hydrogel, the mechanical deformation-sensitive characteristics of the hydrogel were first tested and studied. During the mechanical deformation and recovery cycle of the AD-CD-PNIPAM/rGO hydrogel, the resistance of the hydrogel was recorded in real time to evaluate the sensitivity of the hydrogel. Taking the AD-CD-PNIPAM/rGO hydrogel as an example, the resistance change (around 15–17%) curve of the hydrogel during the mechanical deformation recovery process is shown in Figure 7a–c. And the hydrogel resistance change curve between each deformation and recovery cycle displayed similar results, indicating that the AD-CD-PNIPAM/rGO hydrogel had a stable mechanical deformation-sensitive feature. When mechanical pressure was applied to the hydrogel, the light intensity of the LED bulb could be increased accordingly. After mechanical stress was released, the LED bulb could return to its original light intensity. We further displayed a video in Supplementary Materials (SM) to demonstrate its mechanical deformation-dependent conductivity. The above results indicated that the AD-CD-PNIPAM/rGO hydrogel exhibited a stable mechanical deformation-sensitive property.

In addition, we tested the gauge factor (GF) and cycling tensile sensing ability of the hydrogel. As displayed in Figure S3, the GF was 1.47 when the tensile strain range was 0–50% (R^2^ = 0.99). In addition, the corresponding resistance change hysteresis was approximately 3.1% at 50% strain. This low hysteresis might be because the host–guest interactions and hydrogen bonds rupture during loading, while there is a reform upon unloading. Moreover, polymer chains are straightened and oriented during loading, while chain segments can revert to their original state upon unloading. The cycling sensing stability was assessed by testing the resistance change at 50% strain for 50 cycles. During cycling sensing, the sample disclosed a quite stable resistance change without significant zero drift. The AD-CD-PNIPAM/rGO hydrogel holds great potential for mechanical human movement detection.

Subsequently, the adhesive flexible AD-CD-PNIPAM/rGO hydrogel for human motion monitoring was further evaluated, including finger, wrist, elbow, biceps, and knee movements. The monitoring process of the AD-CD-PNIPAM/rGO hydrogel sensor was described as follows: the AD-CD-PNIPAM/rGO hydrogel was fixed on human tissues and its resistance was recorded by a digital multimeter in real time. Human motion detection test results are shown in Figure 7d–g. The resistance change in the hydrogel was about 7% when the finger was bent, and there was a stable resistance change during each bending recovery process (Figure 7d). The hydrogel also showed a stable resistance change of ~9% when the wrist was bent (Figure 7e). Figure 7f,g show a greater resistance change of ~13% for elbow and muscle movement, because the hydrogel displayed bigger strain changes for elbow and muscle movement. The human motion amplitude difference made the AD-CD-PNIPAM/rGO hydrogel exhibit different resistance change. The above results indicate that AD-CD-PNIPAM/rGO hydrogels have great potential for flexible wearable electronics to detect various human motions in real time.

### 2.7. Cytocompatibility of Hydrogels

**Figure 8:**
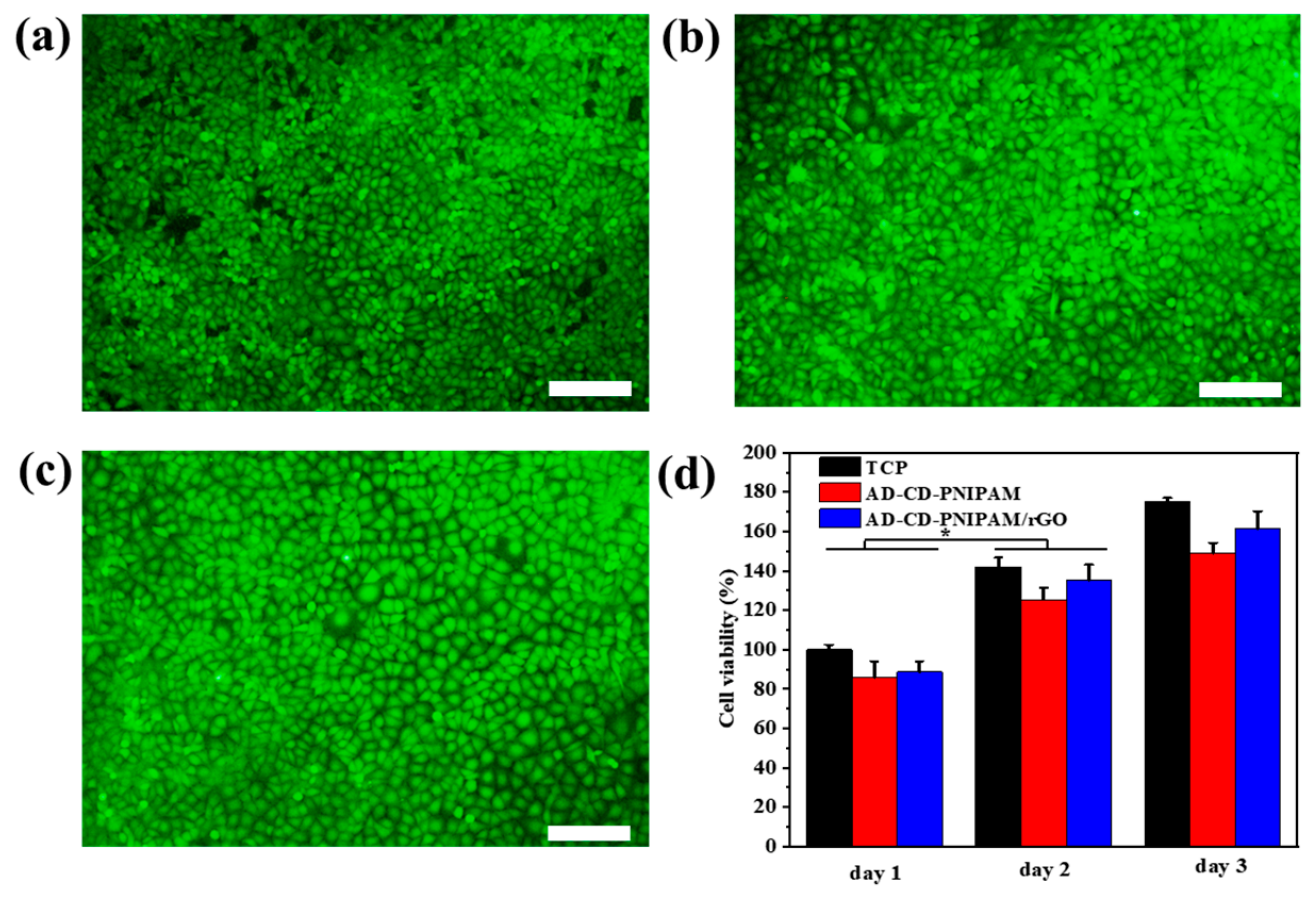
Cytocompatibility of hydrogels. Live/dead images of HUVECs cultured on (**a**) TCP, (**b**) AD-CD-PNIPAM, and (**c**) AD-CD-PNIPAM/rGO groups after 3 days. Scale bar: 200 μm. (**d**) Viability of HUVEC cells for 3 days for TCP, AD-CD-PNIPAM, and AD-CD-PNIPAM/rGO groups. * means *p* < 0.05.

Using HUVECs as model cells, the cytocompatibility of the AD-CD-PNIPAM and AD-CD-PNIPAM/rGO hydrogels was evaluated by the direct contact method. First, the cell morphology (Figure 8a–c) was observed by using live/dead stain reagent when hydrogels and cells were co-cultured on the third day. Viable cells emitted green fluorescence, whereas nonviable cells were identified by red fluorescence. Compared with the hydrogel group, the TCP group exhibited slightly higher green fluorescence and nearly no red fluorescence, and most cells maintained normal morphology.

The Alamar Blue reagent was further employed to assess HUVEC cell viability over three consecutive days. The cell viability results are shown in Figure 8d. After one day of cultivation, the hydrogel group exhibited slightly lower cell viability than the TCP group, indicating that the hydrogels did not adversely affect cell adhesion. After two days of culture, the hydrogel group exhibited cell viability similar to that recorded for the TCP group. Moreover, the viability of each group was significantly higher than that on day one. By the third day, cell viability in the hydrogel group remained similar to the TCP group, further confirming that the hydrogels did not compromise cell viability. Collectively, the live/dead staining test and Alamar Blue test outcomes demonstrated that these hydrogels possess good cytocompatibility, laying a biological foundation for the application in flexible wearable electronic materials.

## 3. Conclusions

In summary, this study successfully develops a multifunctional supramolecular conductive hydrogel that integrates thermal and NIR light responsiveness, self-healing, injectability, stable adhesion to diverse substrates, tunable mechanical properties, and mechanical deformation-sensing capabilities. The key significance lies in the combination of host–guest supramolecular interactions and hydrogen bonding, which enables both structural tunability and dynamic functionality within a single hydrogel system.

In the future, several directions merit further exploration. First, the biocompatibility and long-term stability of the hydrogel should be systematically ameliorated and evaluated to assess its potential for wearable or implantable biomedical devices. Second, the integration of additional stimuli-responsive moieties might enable multi-responsive smart systems for more complex sensing scenarios. Third, optimizing the formulation of hydrogels for scalable manufacturing and improved mechanical durability under cyclic loading will be essential for practical applications. Finally, integrating wireless signal transmission technology could be beneficial for a more intelligent sensing system.

## 4. Materials and Methods 

### 4.1. Materials

Graphene oxide (GO) dispersion solution (10 mg/mL) was provided by XFNANO, Inc. (Nanjing, China). Triethylamine (TEA), N-isopropylacrylamide (NIPAM), acryloyl chloride (AC), and anhydrous *N*,*N*-dimethylformamide (DMF) were acquired from J&K Scientific Ltd., (Beijing, China) and utilized in the as-received condition without additional purification steps. The radical initiator ammonium persulfate (APS) and the accelerator *N*,*N*,*N*′,*N*′-tetramethylethylenediamine (TEMED) were obtained through Sigma Aldrich (Shanghai, China). Any remaining chemicals not explicitly listed met analytical reagent specifications.

### 4.2. Synthesis of Acryloyl-β-Cyclodextrin (AC-CD)

The preparation of AC-CD proceeded as detailed below. An amount of 5 mmol of β-CD was initially dissolved in 20 mL of anhydrous DMF while agitating at room temperature. Next, the reaction was charged with 15 mmol of TEA to scavenge the acid generated during the process. The vessel was subsequently chilled using an ice-water bath, and a separately prepared solution containing 15 mmol of acryloyl chloride dissolved in a further 20 mL portion of DMF was introduced dropwise. The reaction mixture was left to stir over a 24 h interval, during which time the temperature was permitted to rise gradually to ambient levels without applying external heat. Once the reaction had reached completion, the contents were filtered to remove the solid TEA hydrochloride byproduct. The collected filtrate was subsequently poured into chilled acetone to induce precipitation of the product. By adjusting the amount of AC in different batches, AC-β-CD with different grafting ratios of double carbon bonds were obtained.

### 4.3. Synthesis of Acryloyl-1-Adamantanamine (AC-AD)

A solution was prepared by combining 0.4 mmol of 1-adamantanamine and 0.44 mmol of TEA in 40 mL of anhydrous tetrahydrofuran, with the vessel placed in an ice bath. Into this chilled mixture, acryloyl chloride (0.44 mmol) was delivered dropwise. Stirring was maintained for a period of 4 h, allowing the temperature to warm passively to room level. After reaction, the reaction mixture was first filtered to remove precipitates, and the clear filtrate was then concentrated and added to chilled chloroform to induce recrystallization.

### 4.4. Synthesis of AD-CD-PNIPAM and AD-CD-PNIPAM/rGO Hydrogels

Copolymerization of NIPAM with the AC-AD/AC-CD mixture afforded the hydrogels. To prepare AD-CD-PNIPAM, an equimolar blend of AC-AD and AC-CD (10 mg total) together with NIPAM (90 mg) was taken up in 1 mL of ice-cold deionized water to give a chilled homogeneous solution. Into this mixture were introduced 10 µL of a separately cooled APS stock solution (concentration 100 mg/mL) along with 2 µL of TEMED. The components were mixed briefly, after which a 1 mL portion of the resulting precursor was dispensed into 2 mL. Once polymerization was complete, the hydrogel was purified by washing with deionized water. Lyophilization of three individual specimens gave a mean dehydrated mass of 93.7 ± 1.2 mg.

Incorporation of rGO was accomplished by substituting the solvent phase. In this case, the identical quantities of AC-AD/AC-CD (10 mg, equimolar) and NIPAM (90 mg) were taken up in 1 mL of an ice-cooled rGO dispersion. The same volumes of chilled APS (10 µL) and TEMED (2 µL) were then added and homogenized before polymerization. Post-reaction washing with deionized water served as the purification step. Analysis of three freeze-dried replicates revealed an average dry weight of 96.4 ± 3.0 mg for the rGO-containing hydrogel.

### 4.5. Characterizations

^1^H NMR analysis. Proton spectra were recorded using a Bruker Ascend 400 MHz spectrometer (Billerica, MA, USA). Samples of AC-CD (δ (ppm): 3.52–3.68 (m, 28H), 4.78–4.90 (d, 7H), 5.55–5.79 (m, 14H, OH-2 and OH-3), 5.91–6.38 (m, 3H)) and AC-AD (δ (ppm): 1.58−2.10 (m, 15H), 5.46−6.28 (m, 3H), 7.52 (s, 1H)) were dissolved in DMSO-d_6_ prior to analysis. The spatial proximity between protons in the AC-CD/AC-AD complex was probed via two-dimensional NOESY spectroscopy, with the spectrum acquired in D_2_O on the same instrument.

FT-IR analysis. Spectral data were acquired using a Thermo Nicolet 6700 Fourier transform infrared instrument (Waltham, MA, USA). Scans were conducted over the range of 4000–600 cm^−1^, averaging 32 accumulations per spectrum with the resolution set to 4 cm^−1^. All the hydrogel specimens were measured in the dried state.

Volume phase transition temperature determination. The thermal transition behavior of the hydrogels was examined using a TA Q200 DSC instrument (New Castle, DE, USA) under a constant nitrogen purge of 50 mL/min. The samples underwent a programmed temperature profile that included heating from 10 to 50 °C, holding at 50 °C for 3 min, and cooling back to 10 °C. Both the heating and cooling steps were conducted at a rate of 5 °C/min.

Morphological characterization. The internal architecture of the hydrogels was visualized with a Quanta 250 FEG FEI SEM system (Hillsboro, OR, USA) running at an accelerating voltage of 10 kV. Before observation, the frozen hydrogel specimens were sectioned and subjected to lyophilization for a period of 24 h.

Swelling behavior as a function of temperature. Swelling measurements were conducted over 20–45 °C. At each temperature, the samples were blotted with damp filter paper to eliminate surface moisture before recording the wet weight. The swelling ratio was computed as SR = (*W*_s_ − *W*_d_)/*W*_d_, where *W*_s_ represents the mass of the wet gel at a given temperature and *W*_d_ denotes the mass of the dehydrated gel.

Deswelling kinetics at 50 °C. The rate of water expulsion from these hydrogels was monitored by placing the specimens in a 50 °C thermostated water bath. Initially, each gel was permitted to equilibrate fully in deionized water at 25 °C. Once equilibrium swelling had been attained, they were rapidly transferred to the 50 °C bath. At predetermined time intervals, the specimens were retrieved, lightly blotted with filter paper to remove surface moisture, and their mass recorded. The percentage of water retained was determined using the expression: WR = [(*W*_t50_ − *W*_d_)/(*W*_o_ − *W*_d_)] × 100%. In this equation, *W*_t50_ corresponds to the gel mass at a given time point at 50 °C, *W*_o_ is the initial equilibrated mass measured at 25 °C, and *W*_d_ denotes the dehydrated weight.

Photothermal heating evaluation. The hydrogel specimens were subjected to near-infrared illumination using an 808 nm laser source (PSU-III-LED, Changchun, China) positioned 3 cm from the sample surface. The exposure was maintained for a total duration of 10 min. Temperature values were logged at designated time points using a Fluke VT04A visual infrared thermometer (Everett, WA, USA).

Electrical conductivity determination. The hydrogel specimens were sectioned into rectangular slabs (30 × 6 × 0.25 mm^3^). Sheet resistance data were then acquired using a four-probe digital instrument (Agilent B2900A, Santa Clara, CA, USA) fitted with a linear probe assembly having 1.0 mm inter-probe spacing. A constant current of 1 mA was applied throughout. Prior to measurement, the gels were rinsed with deionized water and gently blotted with filter paper to remove surface moisture. Conductivity was subsequently derived from the expression σ = 1/(R_s_ × t), where R_s_ is the recorded sheet resistance and t is the slab thickness.

**Scheme 1:**
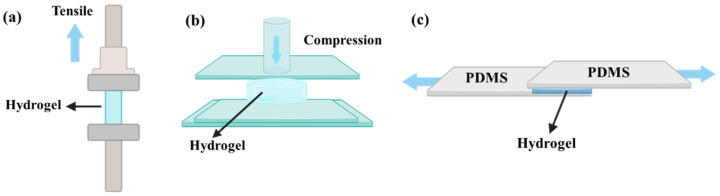
Illustration for mechanical and adhesion testing setups: (**a**) Tensile performance test. (**b**) Compression performance test. (**c**) Lap-shear adhesion performance test.

Mechanical characterization. As depicted in Scheme 1a, tensile testing was conducted on an MTS Criterion Model 43 (Eden Prairie, MN, USA) universal test frame fitted with a 50 N load cell. Rectangular strips having dimensions of 30 mm (length), 6 mm (width), and 0.2 mm (thickness) were prepared from the hydrogel samples. The crosshead was advanced at 5 mm/min to determine both the tensile strength and the elongation at rupture. The samples were cut into cylindrical shapes (approximately 8 mm in height by 10 mm across) and subjected to compressive loading on a DHR-2 rheometer from TA Instruments (New Castle, DE, USA). The compression protocol employed a deformation rate of 6 mm/min and was terminated upon reaching 70% strain.

Adhesion test. A total of 1 mL of the as-prepared hydrogel was employed on various substrates including metal, glass, skin, rubber, and plastic. Then, the adhesive pictures on various substrates were recorded for observation. In addition, the lap shear method was utilized on pig skin or PDMS substrate to evaluate the quantitative adhesive strength (Scheme 1c). Specifically, a hydrogel with a 1 cm (length) × 1 cm (width) size was first placed on the rectangular pig skin or PDMS for one minute. Thereafter, adhesive strength was assessed by pulling the adhered substrate in opposite directions until failure through a mechanical tensile machine (MTS Criterion 43, Eden Prairie, MN, USA).

Rheological characterization. Viscoelastic properties were assessed on a TA DHR-2 rheometer (New Castle, DE, USA) using three testing protocols: (1) Frequency sweep measurements: The storage modulus (G′) and loss modulus (G″) were tracked over an angular frequency range extending from 0.1 to 100 rad/s, with the oscillatory deformation kept at a constant 1%. (2) Amplitude sweep experiments: Crosslinked hydrogel specimens were positioned between parallel plate fixtures at 25 °C. The imposed strain was ramped from 1% up to 400%, with the angular frequency held constant at 10 rad/s. (3) Cyclic step-strain evaluations: Hydrogel disks measuring 20 mm across and 1000 μm in thickness were mounted between 20 mm parallel plates, and the gap was adjusted to 1000 μm at 25 °C. A fixed angular frequency of 10 rad/s was maintained while the strain alternated repeatedly between a low level of 1% and a high level of 400%. Each strain segment was sustained for a duration of 100 s before the switch occurred.

Cytocompatibility assessment. The compatibility of the hydrogels with human umbilical vein endothelial cells (HUVECs) was examined by means of a direct contact method. HUVECs obtained from American Type Culture Collection (ATCC, Mansas, VA, USA) (accession no. PCS-100-013) were maintained in DMEM supplemented with 10% fetal bovine serum, penicillin (100 U/mL), and streptomycin (100 mg/mL). Incubation was carried out in a 5% CO_2_ humidified environment at 37 °C. For testing purposes, the cells were plated into 24-well plates at 8000 cells/cm^2^. Following cell attachment, hydrogel specimens were placed into each well. Metabolic activity as a measure of proliferation was tracked on days 1, 2, and 3 after seeding with the Alamar Blue^®^ reagent (Thermo Fisher Scientific, Waltham, MA, USA). At the designated time intervals, fresh culture medium containing 10% Alamar Blue was introduced, and the plates were incubated for an additional 4 h. Following incubation, 100 μL aliquots of the supernatant were transferred for fluorescence measurement on a SpectraMax plate reader (Molecular Devices, San Jose, CA, USA) with excitation/emission settings of 530 nm and 600 nm, respectively. After three days of cultivation, cell viability was further examined via a live/dead staining procedure. The cell-seeded constructs were first rinsed three times with phosphate-buffered saline (PBS). A staining cocktail consisting of calcein-AM (0.25 μM) and ethidium homodimer-1 (0.5 μM) was added, and the samples were left to stain for 45 min in the dark. Images were then captured on an Olympus IX53 inverted fluorescence microscope. Tissue culture-treated polystyrene (TCP) was employed as the negative control.

Statistical analysis. The data were expressed as mean ± standard deviation. Group comparisons were made using the Student t-test, and *p* < 0.05 was regarded as statistically significant.

## Data Availability

Data will be available upon reasonable request to the corresponding author.

## Supplementary Materials

The following supporting information can be downloaded at https://www.mdpi.com/article/10.3390/gels12050392/s1. Figure S1. Full ^1^H NMR spectrum of (a) AC-AD and (b) AC-CD; Figure S2. Pore size distribution of AD-CD-PNIPAM/rGO hydrogel; Figure S3. Tensile strain sensing behavior of hydrogel. Resistance change at (a) 50% strain. (b) GF within 0–50% strain range. (c) Tensile sensing for 50 cycles at 50% strain; Table S1. Mechanical properties of hydrogels; Table S2. Comparison of supramolecular hydrogels for sensing applications; Movie S1. Mechanical deformation sensing ability of the hydrogel [52,53,54,55,56,57,58,59].

## References

[1] Mo F., Zhou P., Lin S., Zhong J., Wang Y. (2024). A Review of Conductive Hydrogel-Based Wearable Temperature Sensors. Adv. Healthc. Mater..

[2] Liu D., Huyan C., Wang Z., Guo Z., Zhang X., Torun H., Mulvihill D., Xu B.B., Chen F. (2023). Conductive polymer based hydrogels and their application in wearable sensors: A review. Mater. Horiz..

[3] Luo Y., Li J., Ding Q., Wang H., Liu C., Wu J. (2023). Functionalized Hydrogel-Based Wearable Gas and Humidity Sensors. Nano-Micro Lett..

[4] Tao K., Yu J., Zhang J., Bao A., Hu H., Ye T., Ding Q., Wang Y., Lin H., Wu J. (2023). Deep-Learning Enabled Active Biomimetic Multifunctional Hydrogel Electronic Skin. ACS Nano.

[5] Chen Y., Lv C., Ye X., Ping J., Ying Y., Lan L. (2025). Hydrogel-based pressure sensors for electronic skin systems. Matter.

[6] Chen K., Liang K., Liu H., Liu R., Liu Y., Zeng S., Tian Y. (2023). Skin-Inspired Ultra-Tough Supramolecular Multifunctional Hydrogel Electronic Skin for Human–Machine Interaction. Nano-Micro Lett..

[7] Fang K., Wan Y., Wei J., Chen T. (2023). Hydrogel-Based Sensors for Human–Machine Interaction. Langmuir.

[8] Gu Y., Luo Y., Guo Q., Yu W., Li P., Wang X., Ye T., Chang H., Yuan W., Wu H. (2026). Empowering Human-Machine Interfaces: Self-Powered Hydrogel Sensors for Flexible and Intelligent Systems. Adv. Funct. Mater..

[9] Ullah A., Kim D.Y., Lim S.I., Lim H.-R. (2025). Hydrogel-Based Biointerfaces: Recent Advances, Challenges, and Future Directions in Human–Machine Integration. Gels.

[10] Pu J.H., Zhao X., Zha X.J., Bai L., Ke K., Bao R.Y., Liu Z.Y., Yang M.B., Yang W. (2019). Multilayer structured AgNW/WPU-MXene fiber strain sensors with ultrahigh sensitivity and a wide operating range for wearable monitoring and healthcare. J. Mater. Chem. A.

[11] Ul Alam A., Qin Y.H., Nambiar S., Yeow J.T.W., Howlader M.M.R., Hu N.X., Deen M.J. (2018). Polymers and organic materials-based pH sensors for healthcare applications. Prog. Mater. Sci..

[12] Lim H.-R., Kim H.S., Qazi R., Kwon Y.-T., Jeong J.-W., Yeo W.-H. (2020). Advanced Soft Materials, Sensor Integrations, and Applications of Wearable Flexible Hybrid Electronics in Healthcare, Energy, and Environment. Adv. Mater..

[13] Li Z., Lu J., Ji T., Xue Y., Zhao L., Zhao K., Jia B., Wang B., Wang J., Zhang S. (2024). Self-Healing Hydrogel Bioelectronics. Adv. Mater..

[14] Deng Z., Yu R., Guo B. (2021). Stimuli-responsive conductive hydrogels: Design, properties, and applications. Mater. Chem. Front..

[15] Tang S., Feng K., Yang R., Cheng Y., Chen M., Zhang H., Shi N., Wei Z., Ren H., Ma Y. (2025). Multifunctional Adhesive Hydrogels: From Design to Biomedical Applications. Adv. Healthc. Mater..

[16] Wang L., Xu T., Zhang X. (2021). Multifunctional conductive hydrogel-based flexible wearable sensors. TrAC Trends Anal. Chem..

[17] Li G., Li C., Li G., Yu D., Song Z., Wang H., Liu X., Liu H., Liu W. (2022). Development of Conductive Hydrogels for Fabricating Flexible Strain Sensors. Small.

[18] Wu Z., Yang X., Wu J. (2021). Conductive Hydrogel- and Organohydrogel-Based Stretchable Sensors. ACS Appl. Mater. Interfaces.

[19] Gao M., Li J., Zhao S., Li G. (2024). Multi-stimuli-responsive conductive and luminescent hydrogels for wearable sensors and information encryption transmission. Chem. Eng. J..

[20] Li L., Sun X., Guo Y., Cheng W., Shi Y., Pan L. (2025). Recent Advances in Stimuli-Responsive Conductive Hydrogels for Smart Sensing and Actuation: Properties, Design Strategies, and Applications. Macromol. Mater. Eng..

[21] Zhou Y., Li L., Han Z., Li Q., He J., Wang Q. (2023). Self-Healing Polymers for Electronics and Energy Devices. Chem. Rev..

[22] Cerdan K., Thys M., Costa Cornellà A., Demir F., Norvez S., Vendamme R., Van den Brande N., Van Puyvelde P., Brancart J. (2024). Sustainability of self-healing polymers: A holistic perspective towards circularity in polymer networks. Prog. Polym. Sci..

[23] Duceac I.A., Coseri S. (2022). Chitosan Schiff-Base Hydrogels—A Critical Perspective Review. Gels.

[24] Terriac L., Helesbeux J.-J., Maugars Y., Guicheux J., Tibbitt M.W., Delplace V. (2024). Boronate Ester Hydrogels for Biomedical Applications: Challenges and Opportunities. Chem. Mater..

[25] Jin S., Oh D.X., Park J. (2026). Dynamic Disulfide Chemistry for Functional Polymers: Self-Healing, Vitrimer Behavior, and Biochemical/Electronic Applications. ChemSusChem.

[26] Zhang S., Ren D., Zhao Q., Peng M., Wang X., Zhang Z., Liu W., Huang F. (2025). Observation of topological hydrogen-bonding domains in physical hydrogel for excellent self-healing and elasticity. Nat. Commun..

[27] Yang S.-M., Zhou S., Yuan J.-Y. (2025). Self-Healing Elastomers and Coatings via Metal Coordination Bonds. Chem. Eur. J..

[28] Wang S., Ong P.J., Liu S., Thitsartarn W., Tan M.J.B.H., Suwardi A., Zhu Q., Loh X.J. (2022). Recent Advances in Host-Guest Supramolecular Hydrogels for Biomedical Applications. Chem. Asian J..

[29] Liu J., Tan C.S.Y., Yu Z., Li N., Abell C., Scherman O.A. (2017). Tough Supramolecular Polymer Networks with Extreme Stretchability and Fast Room-Temperature Self-Healing. Adv. Mater..

[30] Madl A.C., Myung D. (2021). Supramolecular Host–Guest Hydrogels for Corneal Regeneration. Gels.

[31] Qi Z., Wang Q., Xu H., Lei Y., Li X., Qu D.-H. (2024). Dynamic hydrogel via temporally controlled supramolecular host-guest complex crosslinkers for information self-erasing materials. Mater. Today.

[32] Xu J., Wang K., Li Y., Li Y., Li B., Luo H., Shi H., Guan X., Zhang T., Sun Y. (2023). Injectable Host-Guest supramolecular hydrogel Co-Delivers hydrophobic and hydrophilic agents for enhanced wound healing. Chem. Eng. J..

[33] Tang L., Wang L., Yang X., Fen Y.Y., Li Y., Feng W. (2021). Poly(N-isopropylacrylamide)-based smart hydrogels: Design, properties and applications. Prog. Mater. Sci..

[34] Ansari M.J., Rajendran R.R., Mohanto S., Agarwal U., Panda K., Dhotre K., Manne R., Deepak A., Zafar A., Yasir M. (2022). Poly(N-isopropylacrylamide)-Based Hydrogels for Biomedical Applications: A Review of the State-of-the-Art. Gels.

[35] Deng Z., Guo Y., Zhao X., Du T., Zhu J., Xie Y., Wu F., Wang Y., Guan M. (2022). Poly(N-Isopropylacrylamide) Based Electrically Conductive Hydrogels and Their Applications. Gels.

[36] Zhao Y.S., Lo C.Y., Ruan L.C., Pi C.H., Kim C., Alsaid Y., Frenkel I., Rico R., Tsao T.C., He X.M. (2021). Somatosensory actuator based on stretchable conductive photothermally responsive hydrogel. Sci. Robot..

[37] Yan L.W., Zhou T., Han L., Zhu M.Y., Cheng Z., Li D., Ren F.Z., Wang K.F., Lu X. (2021). Conductive Cellulose Bio-Nanosheets Assembled Biostable Hydrogel for Reliable Bioelectronics. Adv. Funct. Mater..

[38] Xu Y., Rothe R., Voigt D., Hauser S., Cui M.Y., Miyagawa T., Gaillez M.P., Kurth T., Bornhauser M., Pietzsch J. (2021). Convergent synthesis of diversified reversible network leads to liquid metal-containing conductive hydrogel adhesives. Nat. Commun..

[39] Ohm Y., Pan C.F., Ford M.J., Huang X.N., Liao J.H., Majidi C. (2021). An electrically conductive silver-polyacrylamide-alginate hydrogel composite for soft electronics. Nat. Electron..

[40] Deng Z.X., Guo Y., Zhao X., Ma P.X., Guo B.L. (2018). Multifunctional Stimuli-Responsive Hydrogels with Self-Healing, High Conductivity, and Rapid Recovery through Host-Guest Interactions. Chem. Mater..

[41] Li M., Liang Y.P., He J.H., Zhang H.L., Guo B.L. (2020). Two-Pronged Strategy of Biomechanically Active and Biochemically Multifunctional Hydrogel Wound Dressing To Accelerate Wound Closure and Wound Healing. Chem. Mater..

[42] Rezaei F., Damoogh S., Reis R.L., Kundu S.C., Mottaghitalab F., Farokhi M. (2021). Dual drug delivery system based on pH-sensitive silk fibroin/alginate nanoparticles entrapped in PNIPAM hydrogel for treating severe infected burn wound. Biofabrication.

[43] Li H., Li Y.Z., Xu Q., Zhang A. (2020). Multifunctional Clay/PNIPAM Hydrogel Incorporating HxMoO3 Plasmonic Quantum Dot. Energy Environ. Mater..

[44] Zhou Z.X., He Z.R., Yin S.W., Xie X.Y., Yuan W.Z. (2021). Adhesive, stretchable and antibacterial hydrogel with external/self-power for flexible sensitive sensor used as human motion detection. Compos. Part B-Eng..

[45] Won H.J., Ryplida B., Kim S.G., Lee G., Ryu J.H., Park S.Y. (2020). Diselenide-Bridged Carbon-Dot-Mediated Self-Healing, Conductive, and Adhesive Wireless Hydrogel Sensors for Label-Free Breast Cancer Detection. ACS Nano.

[46] Deng Z.X., Hu T.L., Lei Q., He J.K., Ma P.X., Guo B.L. (2019). Stimuli-Responsive Conductive Nanocomposite Hydrogels with High Stretchability, Self-Healing, Adhesiveness, and 3D Printability for Human Motion Sensing. ACS Appl. Mater. Interfaces.

[47] Shuai L.Y.Z., Guo Z.H., Zhang P.P., Wan J.M., Pu X., Wang Z.L. (2020). Stretchable, self-healing, conductive hydrogel fibers for strain sensing and triboelectric energy-harvesting smart textiles. Nano Energy.

[48] He J.H., Shi M.T., Liang Y.P., Guo B.L. (2020). Conductive adhesive self-healing nanocomposite hydrogel wound dressing for photothermal therapy of infected full-thickness skin wounds. Chem. Eng. J..

[49] Wei P.L., Chen T., Chen G.Y., Liu H.M., Mugaanire I.T., Hou K., Zhu M.F. (2020). Conductive Self-Healing Nanocomposite Hydrogel Skin Sensors with Antifreezing and Thermoresponsive Properties. ACS Appl. Mater. Interfaces.

[50] Wang J., Tang F., Wang Y., Lu Q.P., Liu S.Q., Li L.D. (2020). Self-Healing and Highly Stretchable Gelatin Hydrogel for Self-Powered Strain Sensor. ACS Appl. Mater. Interfaces.

[51] Song M.L., Yu H.Y., Zhu J.Y., Ouyang Z.F., Abdalkarim S.Y.H., Tam K.C., Li Y.Z. (2020). Constructing stimuli-free self-healing, robust and ultrasensitive biocompatible hydrogel sensors with conductive cellulose nanocrystals. Chem. Eng. J..

[52] Zhao L., Zhao J., Zhang F., Xu Z., Chen F., Shi Y., Hou C., Huang Y., Lin C., Yu R. (2021). Highly Stretchable, Adhesive, and Self-Healing Silk Fibroin-Dopted Hydrogels for Wearable Sensors. Adv. Healthc. Mater..

[53] Liu L., Li X., Ren X., Wu G.f. (2020). Flexible strain sensors with rapid self-healing by multiple hydrogen bonds. Polymer.

[54] Zhang C., Wang M., Jiang C., Zhu P., Sun B., Gao Q., Gao C., Liu R. (2022). Highly adhesive and self-healing γ-PGA/PEDOT:PSS conductive hydrogels enabled by multiple hydrogen bonding for wearable electronics. Nano Energy.

[55] Zhao L., Li X., Li Y., Wang X., Yang W., Ren J. (2021). Polypyrrole-Doped Conductive Self-Healing Composite Hydrogels with High Toughness and Stretchability. Biomacromolecules.

[56] Wang Q., Pan X., Lin C., Ma X., Cao S., Ni Y. (2020). Ultrafast gelling using sulfonated lignin-Fe^3+^ chelates to produce dynamic crosslinked hydrogel/coating with charming stretchable, conductive, self-healing, and ultraviolet-blocking properties. Chem. Eng. J..

[57] Jia Z., Zeng Y., Tang P., Gan D., Xing W., Hou Y., Wang K., Xie C., Lu X. (2019). Conductive, Tough, Transparent, and Self-Healing Hydrogels Based on Catechol–Metal Ion Dual Self-Catalysis. Chem. Mater..

[58] Wang Z., An G., Zhu Y., Liu X., Chen Y., Wu H., Wang Y., Shi X., Mao C. (2019). 3D-printable self-healing and mechanically reinforced hydrogels with host–guest non-covalent interactions integrated into covalently linked networks. Mater. Horiz..

[59] Xia S., Zhang Q., Song S., Duan L., Gao G. (2019). Bioinspired Dynamic Cross-Linking Hydrogel Sensors with Skin-like Strain and Pressure Sensing Behaviors. Chem. Mater..

