# Supplementary material for: Supramolecular Multiple Stimuli-Responsive Conductive Hydrogel for Flexible Sensing
Source: Gels. 2026 May 2;12(5):392. doi: 10.3390/gels12050392 (PMC13205947; doi:10.3390/gels12050392)
Supplement: Supplementary file 1 [file gels-12-00392-s001.zip › supporting information.pdf]

Supramolecular Multiple Stimuli-Responsive Conductive Hydrogel for Flexible Sensing

Zexing Deng [1,2,*] and Litong Shen [1]

[1] College of Materials Science and Engineering, Xi'an University of Science and Technology, Xi'an 710054, China; 24211225047@stu.xust.edu.cn
[2] Frontier Institute of Science and Technology, Xi'an Jiaotong University, Xi'an 710049, China
* Correspondence: biomaterial@xust.edu.cn

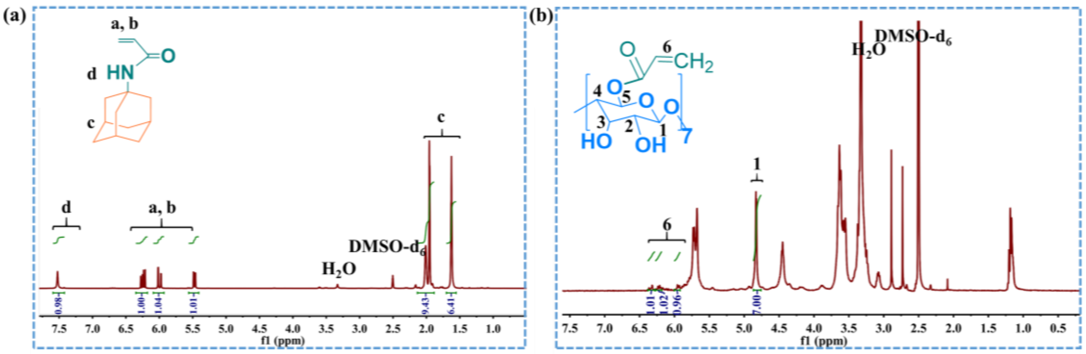

Figure S1. Full ^{1}H NMR spectrum of (a) AC-AD and (b) AC-CD.

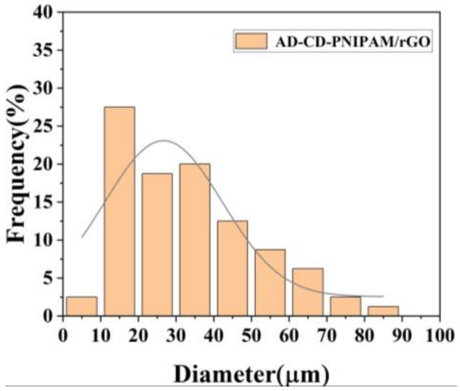

Figure S2. Pore size distribution of AD-CD-PNIPAM/rGO hydrogel.

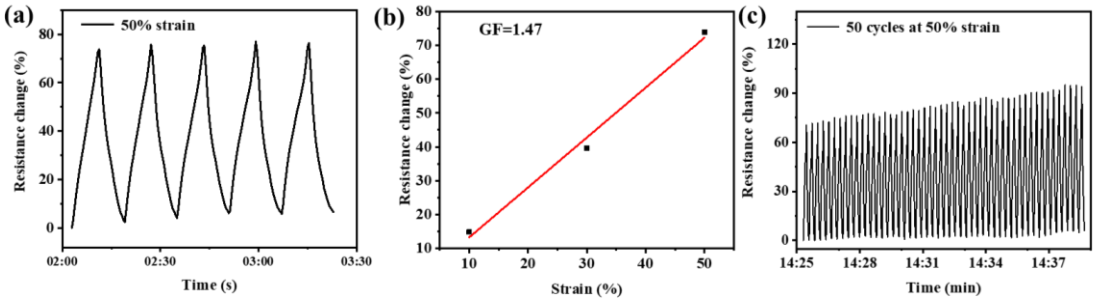

Figure S3. Tensile strain sensing behavior of hydrogel. Resistance change at (a) 50% strain. (b) GF within 0-50% strain range. (c) Tensile sensing for 50 cycles at 50% strain.

Table S1. Mechanical properties of hydrogels.

Hydrogel	Tensile strength (MPa)	Elongation at break (%)	Compressive stress at 70% strain (kPa)
AD-0.5CD-PNIPAM/rGO	0.06±0.01	493±96	5.48±0.24
AD-CD-PNIPAM/rGO	0.14±0.01	464±33	10.46±0.21

AD-1.5CD-PNIPAM/rGO	0.57±0.03	434±39	42.83±1.61

Table S2. Comparison of supramolecular hydrogels for sensing applications.

Materials	Supramolecular interactions	Stimuli-responsivity	Self-healing efficiency	Ref.
SF and P(AM-co-AA)	Hydrogen bond	Tensile strain, compressive stress	~50% with 12 h for tensile strain; ~100% with 2 min for conductivity	1
PAM-co-PHMA and P(St-co-DMAA) microsphere	Hydrogen bond and hydrophobic association	Tensile strain	~58% with 12 h for tensile stress; ~100% with 40 s for conductivity	2
γ-PGA and PEDOT: PSS	Hydrogen bond	Tensile strain	~90% with 24 h for tensile strain; ~100% with 10 s for conductivity	3
PAA, CS, PPy, and Fe^{3+}	Ionic metal coordination	–	~93% with 9 h for tensile stress	4
SL, PAA, and Fe^{3+}	Ionic metal coordination	Potential difference	–	5
TA, DA, TP, and Fe^{3+}	Ionic metal coordination	Potential difference	~85% with 6 h for tensile strain; ~90% with 6 h for tensile stress; ~98% with 6 h for conductivity	6
A-TEG-Ad, β-CD-AOI_2, and GelMA	Host-guest interaction	–	~80% with 1 h for tensile strain	7
AM, LMA, and SDS	Hydrophobic association	Tensile strain, compressive stress	–	8

AC-β-CD, AC-AD, rGO, and NIPAM	Host-guest interaction	Temperature, NIR light, and strain	~84% with 10 s for storage modulus	This work

Abbreviations in this table. SF: silk fibroin; P(AM-co-AA): poly(acrylamide-co-acrylic acid); PAM-co-PHMA/P(St-co-DMAA): polyacrylamide-co-poly(hexadecyl methacrylate)/polystyrene-co-poly(*N,N*-dimethylacrylamide); γ-PGA/PEDOT: PSS: γ-polyglutamic acid/poly(3,4-ethylenedioxythiophene): poly(styrene sulfonate); CS: chitosan; PPy: polypyrrole; SL: sulfonated lignin; TA: tannic acid; DA: dopamine; TP: tea polyphenol; A-TEG-Ad: acryloylated tetra-ethylene glycol-modified adamantine; β-CD-AOI_2: isocyanatoethyl acrylate-modified β-cyclodextrin; GelMA: gelatin methacryloyl.

Movie S1. Mechanical deformation sensing ability of the hydrogel.

References

1. Zhao, L.; Zhao, J.; Zhang, F.; Xu, Z.; Chen, F.; Shi, Y.; Hou, C.; Huang, Y.; Lin, C.; Yu, R.; et al. Highly Stretchable, Adhesive, and Self-Healing Silk Fibroin-Dopted Hydrogels for Wearable Sensors. *Adv. Healthcare Mater.* 2021, *10*, 2101062.

2. Liu, L.; Li, X.; Ren, X.; Wu, G.f. Flexible strain sensors with rapid self-healing by multiple hydrogen bonds. *Polymer* **2020**, *202*, 122657.

3. Zhang, C.; Wang, M.; Jiang, C.; Zhu, P.; Sun, B.; Gao, Q.; Gao, C.; Liu, R. Highly adhesive and self-healing γ-PGA/PEDOT:PSS conductive hydrogels enabled by multiple hydrogen bonding for wearable electronics. *Nano Energy* **2022**, *95*, 106991.

4. Zhao, L.; Li, X.; Li, Y.; Wang, X.; Yang, W.; Ren, J. Polypyrrole-Doped Conductive Self-Healing Composite Hydrogels with High Toughness and Stretchability. *Biomacromolecules* **2021**, *22*, 1273-1281.

5. Wang, Q.; Pan, X.; Lin, C.; Ma, X.; Cao, S.; Ni, Y. Ultrafast gelling using sulfonated lignin-Fe3+ chelates to produce dynamic crosslinked hydrogel/coating with charming stretchable, conductive, self-healing, and ultraviolet-blocking properties. *Chem. Eng. J.* **2020**, *396*, 125341.

6. Jia, Z.; Zeng, Y.; Tang, P.; Gan, D.; Xing, W.; Hou, Y.; Wang, K.; Xie, C.; Lu, X. Conductive, Tough, Transparent, and Self-Healing Hydrogels Based on Catechol–Metal Ion Dual Self-Catalysis. *Chem. Mater.* **2019**, *31*, 5625-5632.

7. Wang, Z.; An, G.; Zhu, Y.; Liu, X.; Chen, Y.; Wu, H.; Wang, Y.; Shi, X.; Mao, C. 3D-printable self-healing and mechanically reinforced hydrogels with host–guest non-covalent interactions integrated into covalently linked networks. *Mater. Horiz.* **2019**, *6*, 733-742.

8. Xia, S.; Zhang, Q.; Song, S.; Duan, L.; Gao, G. Bioinspired Dynamic Cross-Linking Hydrogel Sensors with Skin-like Strain and Pressure Sensing Behaviors. *Chem. Mater.* **2019**, *31*, 9522-9531.
